# Ecological Risks Due to Immunotoxicological Effects on Aquatic Organisms

**DOI:** 10.3390/ijms22158305

**Published:** 2021-08-02

**Authors:** Chisato Kataoka, Shosaku Kashiwada

**Affiliations:** 1 Graduate School of Life Sciences, Toyo University, 1-1-1 Izumino, Itakura, Gunma 374-0193, Japan; 2 Department of Life Sciences, Toyo University, 1-1-1 Izumino, Itakura, Gunma 374-0193, Japan; shokashiwada@gmail.com; 3 Research Centre for Life and Environmental Sciences, Toyo University, 1-1-1 Izumino, Itakura, Gunma 374-0193, Japan

**Keywords:** aquatic toxicology, ecological risk, immune system, immunotoxicity

## Abstract

The immunotoxic effects of some anthropogenic pollutants on aquatic organisms are among the causes of concern over the presence of these pollutants in the marine environment. The immune system is part of an organism’s biological defense necessarily for homeostasis. Thus, the immunotoxicological impacts on aquatic organisms are important to understand the effects of pollutant chemicals in the aquatic ecosystem. When aquatic organisms are exposed to pollutant chemicals with immunotoxicity, it results in poor health. In addition, aquatic organisms are exposed to pathogenic bacteria, viruses, parasites, and fungi. Exposure to pollutant chemicals has reportedly caused aquatic organisms to show various immunotoxic symptoms such as histological changes of lymphoid tissue, changes of immune functionality and the distribution of immune cells, and changes in the resistance of organisms to infection by pathogens. Alterations of immune systems by contaminants can therefore lead to the deaths of individual organisms, increase the general risk of infections by pathogens, and probably decrease the populations of some species. This review introduced the immunotoxicological impact of pollutant chemicals in aquatic organisms, including invertebrates, fish, amphibians, and marine mammals; described typical biomarkers used in aquatic immunotoxicological studies; and then, discussed the current issues on ecological risk assessment and how to address ecological risk assessment through immunotoxicology. Moreover, the usefulness of the population growth rate to estimate the immunotoxicological impact of pollution chemicals was proposed.

## 1. Introduction

Environmental pollution caused by human activities is considered to have become more serious since the industrial revolution and following worldwide industrialization [1]. The scale of polluted areas has sometimes expanded from local scales to global scales [2]. The types and amounts of environmental pollutants have been altered as a result of technological developments and now include pollutants such as mining dust, heavy metals, soot, organochlorine compounds, exhaust gases, wastewater, pharmaceuticals, agricultural chemicals, particulate matters, nanoobjects, and microplastics [1]. When the mechanism responsible for the toxicity of a pollutant is considered, the toxicity is classified as teratogenic [3], carcinogenic [4], neurotoxic [5], or endocrine-disrupting [6] based on the biological effects and chemical properties of the pollutant. Pollutants are known to adversely affect, inter alia, the respiratory [7,8] and cardiovascular systems [9] of aquatic organisms. Organisms have three major biological systems: a nervous system, an endocrine system, and an immune system. These systems regulate respiration, the cardiovascular system, and other biological systems. If these systems are kept in good condition, then organisms can lead normal, healthy lives. Healthy organisms can maintain the populations that compose the biological communities in an ecosystem. Another way to classify the toxicity of a pollutant is therefore to consider the impact of the pollutant on the nervous, endocrine, and immune systems.

To date, there are many reports of pollutant chemicals such as DDT (dichloro-diphenyl-trichloroethane) [10], DES (diethylstilbestrol) and BPA (bisphenol A) [11], heavy metals (mercury [12,13], cadmium [14], copper [14,15], silver [12], aluminum [16], zinc [13]), dioxin [17], organotin compounds [17], pesticides (chlorothalonil [18], *o*,*p*-DDE (dichloro-diphenyl-dichloroethylene) [19], chlorpyriphos [20], and esfenvalerate [20]), and pharmaceuticals [21,22]. Many of these chemicals were known to exhibit neurotoxicity, endocrine-disrupting properties, and teratogenicity, but recent studies have also revealed adverse effects on the immune system. For example, methylmercury attracted attention because its neurotoxicity was the cause of Minamata Disease, but it has also been reported to adversely affect the immune system of bivalve mollusks [23] and gray seals [24]. Some chemicals recognized as environmental pollutants are believed to have immunotoxic effects that were previously hidden by their other, more clearly apparent toxic effects on other biological systems. There are some review papers on fish immunotoxicology. Dunier (1996) have discussed the immunosuppressive effects of pollutants from industry (effluents, heavy metals) and agriculture (pesticides) on freshwater fish as a sentinel model for the aquatic environment [25]. Most of those aquatic pollutants were shown to be partial or total immunosuppressors of the major functions of some freshwater fish immune system. An impairment of humoral, cellular, and/or non-specific immunity compromised the defense mechanisms against pathogens. Segner et al. (2012) published a review about health and biological function as cornerstones of fish welfare and mentioned that good welfare is reflected in the ability of the animal to cope with infectious and non-infectious stressors, thereby maintaining homeostasis and good health, whereas stressful husbandry conditions and protracted suffering will lead to the loss of the coping ability and, thus, to impaired health [26]. Animals’ impaired health would be easier to be infected by pathogens because they are important regulators of their host populations [27,28]. It is known that reproductive activity and immune capacity influence each other, which means, as a result of energy trade-off, costly immunological defenses can impair reproductive function [29,30].

International Council for the Exploration of the Sea has a report that almost all known environmental contaminants seem to have either stimulating or suppressing effects on innate immunity of fish, and there is evidence that the immune system of fish and shellfish reacts to various environmental factors, including natural and anthropogenic ones, and immune responses (either stimulation or suppression) have, therefore, to be considered as an unspecific indicator of environmental stress. However, in the context of infectious diseases, more information is required on effects of contaminants on the acquired immune system and the development of tools for biological effects monitoring and assessment [31]. Recently, Rehberger et al. (2017) have discussed how immune markers were useful to predict adverse changes of fish immunocompetence and disease resistance [32]. The analyzing of a total 241 publications on fish immunotoxicity revealed that there are studies of mainly innate immune responses, non-consisted immune parameters are used, experimental condition are poorly documented, and there is remained insufficient understanding of fish immunotoxicology [32]. Indeed, sufficient understanding of fish immunotoxicology would be crucial to include immunotoxicity in ecotoxicological risk assessment. Also, Segner et al. (2012) discussed in a previous review that immunity is an ecologically relevant trait, which is of key importance for organism survival and population growth against the pressure of pathogens in their environment [33]. Immunocompetence is closely related with fitness parameters such as survival, growth, breeding performance or fecundity [34]. Although, indeed, the immune system is important for organism fitness and population growth, alterations of fecundity and population growth must be an outcome of results which attributed to unbalance of homeostasis through three major biological systems mentioned above. So far only endocrine disruptors are defined to alter fecundity and population growth because there is limited information about biomarkers and mechanisms to explain altered reproduction through other two major biological systems in an integrated manner. Therefore, there is remaining research that needs to understand immunocompetence enough, which is closely related to fecundity through energy-trade-off. The endpoint of ecotoxicological risk assessment would be whether a pollutant chemical can reduce the population size of a target species. Ratio of population growth is a well-recognized parameter to explain the status of the population [35] and would be a good biomarker for ecological risk assessment of pollution chemicals in its ecological context [36,37].

This review aims to introduce ecosystem-relative immunotoxicological studies and biomarkers using aquatic organisms including vertebrates and invertebrates, and then, we can realize that the whole aquatic ecosystem would be threatened by pollutant chemicals through immunotoxic effects; furthermore, the effects may have a potential to reach to population-level. In addition, to assess the risk of immunotoxic pollution chemicals on a population level, we discuss how immunotoxic parameters would be considered.

## 2. The Immune System and Non-Self Substances

The immune system protects higher organisms from non-self substances such as bacteria, viruses, fungi, toxins, and cancer cells that have invaded the organism [38]. The immune system is a defense mechanism that is found not only in vertebrates but also in plants and invertebrates [38]. The immune response is known to reflect the location of the non-self substance and then functions via a complex network of cells, tissues, and organs. As previous reviews have mentioned [26,32,33,39], there is a paucity of information about immunotoxicological effects on aquatic organisms. We hence first summarize general information about immunotoxicology.

The immune system, the nervous system, and the endocrine system require homeostasis to carry out their physiological roles. The nervous and endocrine systems have regulatory roles, and the immune system has a defensive role [40]. Hormones are produced by cells of the immune system, and cytokines, which were thought to be mediators specific to the immune system, have important functions in the endocrine system [41]. Furthermore, an abnormal condition such as an infection brings about responses from the nervous system and the endocrine system through the immune system [42]. In other words, it has become clear that these three major systems function as “one large biological homeostasis maintenance mechanism” [43,44]. This homeostatic mechanism is flexible and can change or evolve according to the life history of each species; it is involved in healthy development, growth, aging, and life, in general, of individual organisms. A modulation or breakdown of this mechanism will adversely affect the health of a single individual and may impact an entire population if it occurs in many individuals simultaneously. Exposure to environmental pollutants can be a factor in the modulation and/or disruption of systems that maintain homeostasis. For example, snake toxins [45] and alcohol [46] are chemical substances that have been used to affect the nervous system since prehistoric times, and DES has been used in medical interventions to affect the endocrine system since the 1960s [47]. Allergies due to natural toxins and foods have been known to be caused by substances that affect the immune system [48]. The state of health maintained by the normal interactions between the nervous, endocrine, and immune systems may be easily disrupted by the failure of any of these systems. In particular, the immune system plays a central role in biological defense against pathogens [38,49]. Chemically induced abnormalities in the immune system may therefore have side effects such as increased susceptibility to pathogens. Furthermore, the side effects may impact not only individuals but also populations, communities, and entire ecosystems.

When a pathogen invades an organism, the organism activates its immune system to eliminate the pathogen. However, if the immune system remains activated after the pathogen has been eliminated, the organism will damage its own cells [50]. Organisms therefore usually have a mechanism that activates the immune system and a mechanism that suppresses it, and the immune system is controlled appropriately by their interaction. Genetic or environmental factors may modulate or disrupt this control mechanism or cause it to function abnormally. These effects can be roughly divided into abnormal enhancement and suppression of immunity. The former is a factor in the exacerbation of allergies [51] and autoimmune diseases [52,53], and the latter is a factor that contributes to immunodeficiency [53,54], which causes a decrease of host resistance to pathogens and tumor cells. It has become clear that immunodeficiency due to genetic factors (for example, deficiency of phagocytic cells, complements, T cells, or antibodies involved in biological defense) contributes to an increase in susceptibility to infection by various pathogens [55]. However, immunodeficiency due to environmental factors (for example, pH, temperature, ultraviolet rays, a nutritional deficiency, or environmental pollutants) is thought to occur not as the result of an effect on specific immunocompetent cells, tissues, or organs, but rather as the result of an imbalance between the mechanisms that activate and suppress the immune system [16,56,57,58,59,60,61]. The immune system, therefore, contributes to the maintenance of homeostasis only when it is appropriately controlled by the interaction between immunocompetent cells, tissues, and organs. Hence, exposure of organisms to chemical pollutants (particularly immunotoxic chemicals) can induce multiple, simultaneous impacts on various cells, tissues, and organs under the control of the immune system [13,43,62,63,64,65,66].

Toxic chemicals are among the environmental factors that affect the immune system. Studies of the effects of toxic chemicals on the immune system began to be reported in the 1970s, when exposure to heavy metals was reported to increase the susceptibility of experimental organisms to infections [67,68], and exposures to PCBs (polychlorinated biphenyls) and dioxins were found to cause pathological changes in lymphoid tissues [17]. The immune system can be a target of toxic chemicals. Abnormalities of the immune system induced by drugs and environmental pollutants are called “immunotoxicity”, and the corresponding research area has been called “immunotoxicology” [69]. Many of the initial immunotoxicological studies evaluated the effects of toxins such as anticancer agents, dioxins, DDT, PCBs, organotin compounds, lead, and cadmium on humans, domestic animals, and laboratory animals (mice, rats, and rabbits). It has been reported that these chemicals increase the susceptibility of organisms to pathogens. Vos [17] has warned that chemical pollutants from industrial waste could reduce the immune response of aquatic organisms and increase their susceptibility to infection. In fact, striped dolphins with high levels of PCBs in their blubber and liver died in large numbers during a morbillivirus epidemic [70], and harbor seals with organochlorine compounds in their blubber died during an epidemic of seal distemper virus [71]. Results of recent immunotoxicological studies have been used prophylactically (for risk aversion) in tests of the toxicity of new drugs. In 2006, the Japan-US-European Union International Council for Harmonization of Pharmaceutical Regulations established “Guidelines for Immunotoxicity Testing of Drugs” [72], and in 2007, the European Union Pharmaceutical Examination Agency established “Guidelines for Evaluation of Immunogenicity of Biopharmacy” [73]. The usefulness of immunotoxicological evaluations to screen new drugs for biotoxicity is recognized worldwide.

## 3. Immunotoxicological Research as Aquatic Ecotoxicology

A few immunotoxicological studies have been conducted using aquatic organisms to understand the immunotoxic effects of chemical pollutants on those organisms (Table 1, Table 2 and Table 3). Immunological research on humans is very advanced compared to similar research on other organisms. Understanding of the immune systems in non-humans has been based on a comparative-biological approach. The bodies of organisms have evolved into complex structures, and immune systems have evolved as well, but organisms have a common ancestor with a simple morphology. Therefore, immunotoxicological studies have been conducted using the same immunotoxicological evaluation methods, regardless of the species.

### 3.1. Invertebrates

Invertebrates have simple and primitive immune systems, and they have simpler body structures than vertebrates. Although the details of the biological defense mechanisms of invertebrates have not been clarified, they do not have an acquired immune system such as a lymphatic system, and they do not produce antibodies [74,75,76]. The biological defense of invertebrates is based on innate immunity [75,76]. Invertebrates prevent pathogens from invading their bodies through physical and chemical barriers such as the epithelium, shell, and mucous layer. Pathogens that gain entry are eliminated via phagocytosis by the coelomocytes circulating in the body [76,77], the secretion of antibacterial lectin [76,78], the secretion of the antibacterial enzyme lysozyme [76,79], the production of cytokines that induce inflammatory reactions [76,80], and cell–cell communication through prophenoloxygenase and eikosanoid [76]. Immunotoxicological tests on chemicals such as antibiotics, pharmaceuticals, fungicides, and copper have been conducted using mussels, oysters, and ascidians [15,18,21,22]. Bivalves are among the aquatic invertebrates often used in immunotoxicological research. They are probably used because it is relatively easy to collect coelomocytes, which are the main cells responsible for the immune response in invertebrates, and bivalves are highly valued in the commercial bivalve fishery. There are reports of decreases in the number and functionality (phagocytic activity) of the coelomocytes of these organisms due to exposure to chemical substances [15,18,21,22]. In addition, a study using the polychaete *Eurythoe complanata* has found that the viability and functionality of its coelomocytes decrease within four hours of copper exposure [81].

### 3.2. Vertebrates

Fish, amphibians, and marine mammals are known as aquatic vertebrates with complex immune systems. Fish are classified as either jawless or jawed, and jawed fish are further classified as either cartilaginous or teleost [82]. In organisms classified as jawless, there is an absence of both lymphoid tissues (in the thymus, spleen, gut-associated lymphoid tissue, kidneys, and liver) and antigen-specific molecules (immunoglobulin [Ig], T cell receptor [TCR], major histocompatibility complex [MHC] classes I and II, and complementary systems (lectin pathway, classical pathway, lysis pathway)); furthermore, there is no mechanism to reject allogeneic transplants [82,83]. However, sharks and rays, which are cartilaginous fish (Gnathostomata), have independent lymphatic organs such as the thymus and spleen, and both of their humoral and cell-mediated immune responses are as well developed as those of higher vertebrates [84]. In addition, molecules involved in specific antigen recognition such as Ig, MHC, and TCR are also functionally and structurally differentiated to almost the same level as they are in mammals [85]. A dramatic change is thus thought to have occurred in the biological defense system when the fish with jaws diverged from the jawless fish [44]. Because of these evolutionary adaptations, cartilaginous fish are not the subject of immunotoxicological research but rather the subject of research on the origin and evolution of the immune system from a comparative biological perspective [86,87]. In addition, although it is difficult to collect and breed test organisms, there have been a few examples of immunotoxicological studies targeting cartilaginous fish.

Amphibians have a more developed immune system than invertebrates and fish [88]. A biological defense mechanism common to fish is the secretion of antibacterial proteins from the epidermis and digestive tract [89]. Phagocytic cells such as macrophages and neutrophils, NK cells, cytokines, MHC class I and II, T cells, and B cells are all found in fish and amphibians [88,90]. In both fish and many amphibians, the thymus and spleen serve as central and terminal lymphoid organs involved in lymphocyte maturation, but fish and amphibians do not have the lymph nodes and lymphoid bone marrow found in humans [91]. A biological defense mechanism developed more in amphibians than in fish is the presence of the immunoglobulin isotype IgY, which is equivalent to mammalian IgG. IgY and IgG have the function of promoting phagocytosis by phagocytic cells and promoting the degradation of extracellular microorganisms and toxins. Mass mortality and population extinction for unknown reasons of the amphibian Rana muscosa have been occurring over the last half-century, and the phenomenon has been seen on a global scale across continents [92]. One of the various hypothesized causes of this mass mortality and extinction is thought to be that immunosuppression due to radiation and low-temperature conditions decreased immunity of amphibians due to pollutants such as pesticides, and increased susceptibility to pathogens [93,94,95,96,97]. There have been few reports, however, of ecotoxicological, immunotoxicological studies using amphibians. Christin et al. [98] have investigated the effects of pesticides on the immune systems of Xenopus laevis and Rana pipiens in terms of splenocyte survival, phagocytosis, lymphopenia reaction, and cell densities; they have reported that pesticides disrupt the phagocytosis of Xenopus-derived splenocytes and suppress the lymphoblastization reaction of Xenopus-derived splenocytes [98]. That report has also shown that the amphibian immune system is also a target of toxic environmental pollutants.

**Table 1:** Chemical substances that have been reported to have immunotoxic effects in invertebrates (bivalves and colonial ascidians).

Classification		Organism	Chemicals	Condition	Reported Impacts	Reference
Invertebrates	Bivalve	*Eurythoe complanata*	Cu	Lab	Increase in phagocytosis	[81]
		*Crassostrea virginica*	Tributyltin	Lab/in vivo	Increased protozoan infection rate	[99]
		*Mya arenaria*	Hg	Lab/in vivo	Decreased phagocytosis	[100]
			Cu	Lab/in vivo	Decreased superoxide production Stimulation of phagocytic activity Reduction of a percentage of hemocytes showing binding of lectins	[15]
		*Cyrtodaria siliqua*, *Mactromeris polynyma*, *Mesosdesma arctatum*, *Mya arenaria*, *Mya truncata*, *Mytilus edulis*, *Serripes groenlandicus*, *Siliqua costata*, *Dreissena polymorpha*, *and Elliptio complanata*	Ag, Cd, Hg, and Zn	Lab/in vivo	Low doses of mercury (organic and inorganic) and Zn suggest a hormesis-like stimulation of phagocytic activity At higher levels of exposure, all metals tested induced a significant dose-related inhibition of hemocyte phagocytosis	[23]
		*Mytilus galloprovincialis*	polystyrene nanoplastics	Lab/in vivo	Decrease in phagocytic activity	[65]
		*Crassostrea gigas*	chlorothalonil	Lab/in vivo	No effects on phagocytosis	[18]
		*Elliptio complanata*	Antibiotics	Lab/in vivo	Increased phagocytic activity Increased ROS production	[22]
	Colonial ascidian	*Botryllus schlosseri*	Ibuprofen	Lab/in vivo	Reduction in both phagocytic activity and lysosomal membrane stability	[21]

**Table 2:** Chemical substances that have been reported to have immunotoxic effects in teleost fish.

Classification		Organism	Chemicals	Condition	Reported Impacts	Reference
Vertebrates	Teleost fish	*Oncorhynchus mykiss*	Cd	Lab/in vivo	Decreased phagocytosis Lymphocyte dysfunction Decrease in antibody production Decreased lysozyme activity	[13]
			Cu, Al, and Cd	Lab/in vivo	Effects on induction of chemiluminescent response in phagocytes	[14]
			Retene (PAH)	Lab/in vivo	Increased white blood cell count Increased antibody production Increased expression of immune-related genes	[62]
			TCDD	Lab/in vivo	Suppression of the induced response of splenic lymphocytes to pokeweed mitogen	[101]
		*Anguilla anguilla*	Cu	Lab/in vivo	Increased bacterial infection rate	[102]
		*Oncorhynchus tshawytscha*	*o*,*p*-DDE	Lab/in vivo	Reduction in the ability of splenic leukocytes.	[19]
			chlorpyrifos and esfenvalerate	Lab/in vivo	Decrease of cytokine expression	[20]
		*Oryzias latipes*	benzo[a]pyrene (PAH)	Lab/in vivo	Suppressed mitogen-stimulated T- and B-lymphocyte proliferation Reduction in the phagocyte-mediated radical ⋅O_2_^−^ production	[103]
			Ni	Lab/in vivo	Elevated intracellular⋅O_2_^−^ production by kidney phagocytes Reduction in extracellular ⋅O_2_^−^ production	[61]

**Table 3:** Chemical substances that have been reported to have immunotoxic effects in amphibians and marine mammals.

Classification		Organism	Chemicals	Condition	Reported Impacts	Reference
Vertebrates	Amphibians	*Xenopus laevis*	Atrazine, metribuzine, endosulfan, lindane, aldicarb, and dieldrin	Lab/in vivo	Increased phagocytic activity	[98]
		*Rana pipiens*	Atrazine, metribuzine, endosulfan, lindane, aldicarb, and dieldrin	Lab/in vivo	Lymphocyte dysfunction	[98]
		*Xenopus laevis*	Hg	Lab/in vivo	Decreased phagocytosis	[12]
	Marine mammals	*Callorhinus ursinus*	PCBs, chlorinated pesticides	Field/in vitro	Increase in neutrophil count	[104]
		*Phoca vitulina*	PCBs	Field/in vitro	Decreased phagocytosis Increased frequency of respiratory bursts Lymphocyte dysfunction	[63]
			PCBs, Dioxins	Field/in vitro/in vivo	in vivo delayed-type hypersensitivity (DTH) reactions correlated well with in vitro tests of T-lymphocyte function	[105]
			Environmental contaminants	Field/in vitro/in vivo	Lower natural killer-cell activity and mitogen-induced proliferative T -cell responses	[106]
		*Tursiops truncatus*	Hg	Field/in vitro	Increase in neutrophil count Decrease in lymphocyte count	[64]
		*Phocoena phocoena*	PCBs, PBDE, *p*,*p’*-DDT, *p*,*p’*-DDE	Field/in vitro	Thymic atrophy and splenic depletion, and lymphoid depletion	[107]
		*Halichoerus grypus*	Hg	Field/in vitro	Mercury decreased the immune response. Phagocytosis is more affected by MeHgCl.	[24]

Large aquatic vertebrates such as marine mammals (e.g., seals, dolphins, and whales) have immune systems [108,109]. The lifespan of marine mammals is relatively long compared to other marine organisms, and because they are near the top of marine food chains, they are easily exposed to high concentrations of environmental pollutants that bioaccumulate through the food chain. Because they have a high-fat content, they are more prone to accumulate fat-soluble pollutants (e.g., PCBs) in their bodies than other aquatic organisms. There is hence the concern about the biological effects of the high concentrations of chemical pollutants that have accumulated in their bodies. Dietz et al. (1989) have reported the deaths of 18,000 dolphins due to outbreaks of morbillivirus in Europe in 1988 [110], and Aguilar et al. (1993, 1994) have reported the deaths of 1000 dolphins due to outbreaks of morbillivirus in the Mediterranean between 1990 and 1992 [70]. Whether these outbreaks were associated with environmental pollutants remains unclear, but it is possible that disorders of immune systems caused by environmental pollutants may have caused these mass mortalities. In addition, high concentrations of PCBs (10–500 μg/g wet weight) have been found in the tissues of a dead Beluga whale that lived in the estuary of the St. Lawrence River, which had been contaminated with organic chemicals such as organochlorine compounds and polycyclic aromatic hydrocarbons for more than 50 years [111]. In addition, a Beluga whale was reported to have tumors, pneumonia, and inflammation that affected its mammary gland [112]. Ross et al. (1995) have conducted a 2.5-year breeding experiment on harbor seals to investigate the effects of dietary contaminants on their immune function [105]. Comparison with a control group (fed relatively less polluted fish from the North Atlantic) revealed a significant increase in white blood cell counts (lymphocytes), activation of NK cells, and a significant decrease of the function of lymphocytes in the exposed group (fed contaminated fish from the Baltic Sea) that suggested that the immune function of the seals in exposed areas may have been impaired [105,106,113].

Individual marine mammals have rarely been studied because of the difficulty of breeding them. In many cases, samples taken from wild marine mammals have therefore been used for testing. In many cases, lymphocyte differentiation involved in immune function has been used as a metric to evaluate the status of their immune systems [114,115]. Other biomarkers of immunotoxicity have included histological evaluation of immune tissues (e.g., bone marrow, thymus, spleen, lymph nodes, mucosa-associated lymphoid tissues), abundance of immunocompetent cells, phagocytosis, respiratory bursts, cellular NK activity, antibody production, and expression of cytokine genes [116]. Most of the immune system of marine mammals is common to that of humans, and marine mammals have both innate and acquired immunities. Changes in maternal nutritional status due to pregnancy and childbirth are also thought to be associated with changes in immune function and seasonal viral epidemics [117]. The range of annual fluctuations in the immune system of marine mammals is therefore quite large.

## 4. Biomarkers for Immunotoxicological Studies and Their Application to Aquatic Toxicology

The immune system has developed along with the evolution of living organisms as a biological defense mechanism [118]. There are hence commonalities among the innate immune systems developed by invertebrates, fish, amphibians, and mammals in the early stages of evolution (Table 4, Table 5 and Table 6) [119]. Lectins, anti-microbial proteins, and phagocyte are shown in aquatic invertebrate and vertebrate organisms; therefore, they are thought of as primitive innate immunity. Along with biological evolution, there is more variety of complement systems (Table 4). In innate immunity, phagocyte that prey on pathogens induce an inflammatory response, and then respiratory bursts are induced as the response of cellular biodefense against pathogens. Moreover, natural killer (NK) cells in lymphoid tissue work in innate immunity. NK cells kill virus-infected cells, and secrete cytokines that regulate the inflammatory reactions [49,120]. Lymphocytes (T cells, B cells, and NK cells) mature in lymphoid tissue and contribute to a specific antigen as an antibody in acquired immunity (Table 5 and Table 6). During the immune response, the lymphoid tissue is affected and the histological information is important to understand the immune response [121,122,123]. The method of evaluating the immune function of humans is often applied to the immunotoxicological evaluation of aquatic organisms. We will thus introduce the biomarkers used for aquatic immunotoxicological evaluation. Proper use of those biomarkers is very important for clinical understanding the mechanism of the response of living organisms to drugs [124]. However, because the measurement of biomarkers is premised on the assumption that the organism is healthy, it is not possible to measure biomarkers using an organism if the responsiveness of the organism has been reduced by the toxicity of a drug. Similarly, when immunotoxicological tests are performed on unhealthy organisms, the immune system may not function properly, and the effects of immunotoxicity may be underestimated or overestimated. The immune system was originally a mechanism that functioned properly in healthy organisms and protected the organism from non-self substances. Attention must also be paid to the history of chemical exposure, especially when conducting immunotoxicological studies on wildlife.

### 4.1. Phagocytic Activity

An increase of phagocytic activity is an index used to evaluate the activity of the immune system and is one form of cellular biological defense among the biological defense mechanisms classified as innate immunity. Aquatic species used in the phagocytic activity test have included a polychaete (*Eurythoe complanata*), clams (*Cyrtodaria siliqua*, *Mactromeris polynyma*, *Mesosdesma arctatum*, *Mya arenaria*, *Mya truncata*, *Serripes groenlandicus*, and *Siliqua costata*), the blue mussel (*Mytilus edulis*), a freshwater mussel (*Elliptio complanata*), an oyster (*Crassostrea gigas*), the American plaice (*Hypoglossoides platessoides*), the mummichog (*Fundulus heteroclitus*), a colonial ascidian (*Botryllus schlosseri*), the zebrafish (*Danio rerio*), the rainbow trout (*Oncorhyncus mykiss*), an amphibian (*Xenopus laevis*), the grey seal (*Halichoerus grypus*), pups of the northern fur seal (*Callorhinus ursinus*), and the bottlenose dolphin (*Tursiops truncatus*) [12,13,14,15,18,21,22,23,24,65,81,98,100,101,104,125,126]. Cellular biological defense is carried out by macrophages, neutrophils, and dendritic cells, all of which are white blood cells [38,49]. All of these cells use phagocytosis to prey on and digest foreign substances such as pathogens that have entered the body. Phagocytosis by phagocytic cells is one of the biological defense mechanisms commonly found in vertebrates and invertebrates, and assessment of phagocytic activity is a highly versatile immunotoxicological evaluation method. Phagocytes not only attack foreign substances by phagocytosis but also activate T cells by “antigen presentation”, which conveys information about foreign substances to T cells [121]. They also initiate the production of antibody signals that are related to the activation of acquired immunity.

### 4.2. White Blood Cells

Leukocytes are immunocompetent cells involved in both innate immunity and acquired immunity [127,128]. Because leukocytes play a central role in immune response, their numbers increase drastically in response to the presence of pathogens or drugs in an organism [38]. Factors such as infectious diseases, inflammation, allergic diseases, and malignant tumors are known to increase white blood cell counts, and anticancer agents and irradiation are known as factors that decrease white blood cell counts [38,49]. In humans, immunocompetent cells include neutrophils, eosinophils, basophils, monocytes, T cells, and B cells, the abundance of which vary depending on specific factors. Measurements have been made of the concentrations of leukocytes in the blood of chinook salmon (*Oncorhynchus tshawytscha*) exposed to *o*,*p*-dichlorodiphenyldichloroethylene (*o*,*p*-DDE) [19] and of rainbow trout exposed to the alkyl polycyclic aromatic hydrocarbon (PAH) retene (7-isopropyl-1-methyl phenanthrene) [62].

### 4.3. Respiratory Bursts

The evaluation of respiratory bursts uses the response of cellular biodefense factors as an index of antibacterial activity among the biodefense mechanisms classified for innate immunity [22,61,103,129]. When inflammation occurs, the activities of processes such as xanthine oxidase in phagocytic cells and the vascular endothelium, phospholipase A2 regulation of arachidonic acid mobilization, and the activity of intracellular mitochondria produces more hydrogen peroxide, nitric oxide, and reactive oxygen species (ROS), which have strong oxidizing action and antibacterial activity [130]. This phenomenon is called a respiratory burst [131,132]. Respiratory bursts play a central role in bactericidal action in neutrophils, and the oxidizing action is therefore used as a metric of immunotoxicity [131]. Due to their high reactivity, ROS, which has a bactericidal effect, impact surrounding cells other than pathogens, and they are among the factors that cause lipid damage due to peroxidation, protein damage, and cell damage through damage to DNA [133,134,135]. Redox enzymes such as superoxide dismutase and catalase as well as low-molecular-weight antioxidants maintain the balance in a healthy living body by removing ROS [136,137,138]. To elucidate the possible relationship between environmental contaminants and increased disease susceptibility of aquatic organisms, the oxidizing action of ROS has been measured using bivalves (*Mytilus edulis*, *Mytilus galloprovincialis*, and *Crassostrea gigas*) *and* medaka (*Oryzias latipes*) [15,18,61,65,103,129,139].

### 4.4. NK Cell Activity

Natural killer (NK) cells are cytotoxic lymphocytes that are among the cellular biodefense factors in innate immunity [140]. Activated NK cells through innate immune reaction enter infected tissues during viral infections, kill virus-infected cells, secrete cytokines to prevent the spread of infection, and delay the growth of the viruses inside infected cells. NK cells also recognize and kill some tumor cells. Individuals deficient in NK cells cannot eliminate a virus even though their acquired immunity works normally [141]. The activity of NK cells is used as an index to evaluate the biological defense of an organism against viruses. NK cell activity has been reported to be significantly lower in harbor seals fed fish from the highly polluted Baltic Sea versus the relatively unpolluted Atlantic Ocean [106].

### 4.5. Cytokines

Cytokines are low-molecular-weight proteins released from mainly immunocompetent cells and responsible for their signal transduction [142]. There are thought to be several hundred types of cytokines. Cytokines are involved in both innate and acquired immunity as well as in the control of immune responses. Cytokines are roughly classified based on whether they promote inflammatory reactions or inhibit inflammatory reactions. When a pathogen invades the body, immunocompetent cells are summoned to the infected tissue, and their action prevents the spread of the infection. This “convocation” is one of the phenomena that occur as a result of cytokine signal transduction, and because it is involved in immune response, it is frequently used as a biomarker for immunotoxicological research; cytokines are quantified mainly via an enzyme-linked immunosorbent assay or a quantitative polymerase chain reaction [142]. In a study of the expression of immunoregulatory genes following exposure of juvenile Chinook salmon to pesticides, the expression of cytokines was observed to have been altered [20]. One metric of ecosystem health has been the level of cytokines in harbor seals exposed to increasing stress caused by anthropogenic activities that impacted their marine environment [127].

### 4.6. Lymphocyte Blastization

The lymphocyte blastization reaction test uses the reaction of the biological defense mechanism as an index to classify acquired immunity [61]. Because the response in terms of acquired immunity is used as an index, the organisms to be tested are limited to teleost fish, amphibians, and mammals. After differentiating from hematopoietic stem cells, lymphocytes (T cells, B cells, and NK cells) mature in lymphoid tissues and exert their respective functions [121,122,123]. In the steady-state, mature lymphocytes do not divide or proliferate anymore, but when they encounter a specific antigen, they take a morphologically premature form (immature cell morphology) and proliferate by cell division [121,122,123]. This phenomenon is called blast formation (juvenile) and is used to evaluate the activity of lymphocytes. Aquatic pollutants affect the activity of lymphocytes. The proliferation of lymphocytes in frogs (*Rana pipiens*) [98], medaka (*Oryzias latipes*) [61], and rainbow trout [101] have been assessed after exposure to pollutant chemicals. The counts of lymphocytes in harbor seals (*Phoca vitulina*) [63,105] and bottlenose dolphins (*Tursiops truncatus*) [64] have likewise been used to assess the effects of chemical and biological pollution. Juvenile grey seals have been used to quantify the immunotoxicological effects of mercury in the St. Lawrence Estuary. In that study, methylmercury chloride was shown to decrease lymphoblastic transformation responses in vitro [24]. The mitogen-induced proliferative T-cell responses of harbor seals have been found to be significantly reduced when the seals are fed fish from polluted waters [106]. Examination of the effects of organochlorine contamination on the immune systems of harbor porpoises has revealed that thymic atrophy and splenic depletion are significantly correlated to increased levels of PCBs and PBDE in their tissues [107].

### 4.7. Antibody-Producing Cells and Antibody Volume

Antibodies are responsible for the recognition of antigen-specific molecules in the acquired immune system. Whereas non-specific biological defense reactions are carried out in the innate immune system, immunocompetent cells carry out a specific biological defense reaction against a pathogen marked with an antibody to more efficiently control the pathogen [143]. The increased amounts of immunoglobulin and a number of antibody-producing cells are therefore used as evaluation indexes of acquired immunity. However, because antibodies for each species are required for their detection, there are few examples of the use of aquatic organisms for immunotoxicological testing [103,125]. Organisms that have been tested have been limited to teleosts, amphibians, and mammals. The effect of the PAH retene on rainbow trout has been examined by co-injecting the trout with retene and formalin-killed *Aeromonas salmonicida*; measurements revealed an overall increase of the titer of the *A. salmonicida*-specific antibody [62]. 

### 4.8. Histology of Lymphoid Tissue

Histological verification of lymphoid tissues (such as bone marrow, thymus, spleen, lymph nodes, and mucosa-related lymphoid tissues), which are important tissues in acquired immunity, is also used for immunotoxicological assessment [107,144,145]. Because lymphoid tissue is present in only highly developed organisms, the organisms tested are limited to teleost fish, amphibians, and mammals. The primary lymphoid tissues, bone marrow, and the thymus gland are places where lymphocytes differentiate and mature to the stage where they can respond to pathogens, whereas the secondary lymphoid tissues, such as the spleen, lymph nodes, and mucosa-related lymphoid tissues, are tissues in which mature lymphocytes are activated in response to pathogens that have invaded the body [121,122,146]. Thymic and spleen dysfunction and thymic atrophy due to exposure to PCBs and dioxins have been reported in mice, harbor seals, and northern fur seals [17,63,104]. 

### 4.9. Resistance to Pathogens

Although human-centered immune research is highly developed, no quantitative marker of immune function has yet been found. Although immunotoxicological assessment via biomarker measurements can detect “variations in the immune system”, it does not directly identify the effect of such variations on the health of individual organisms. However, related studies have been used to evaluate the resistance of individual organisms such as medaka and zebrafish to bacterial, viral, or parasitic pathogens in terms of the percentage of survivors during any period of time [103,126].

**Table 4:** Comparison of innate immune systems among aquatic organisms.

Phylum	Class		Examples	Innate Immunity					References
				Lectins, Antimicrobial Proteins	Phagocyte	Complement System			
						Lectin Pathway	Classical Pathway	Alternative Pathway	
Invertebrate	crustacean		polychaete, crab, shrimp	◎	◎	○	NE	NE	[81,147,148,149,150]
	shellfish		bivalves	◎	◎	○	NE	NE	[12,14,15,22,23,65,100]
Vertebrate	jawless fishes	cyclostomes	hagfish	◎	◎	◎	NE	NE	[151,152,153,154]
			lamprey eel	◎	◎	◎	NE	NE	[154,155]
	jawed fishes	cartilaginous fishes	sharks, rays	◎	◎	◎	◎	◎	[156,157,158]
		osteichthyans	trout, flounder, medaka, zebrafish, mummichog, carp	◎	◎	◎	◎	◎	[12,13,14,19,62,101,103,126]
		amphibian	newt	◎	◎	◎	◎	◎	[159,160,161]
			frog	◎	◎	◎	◎	◎	[12,98,162,163]
		mammal	Mouse, dolphin, seal, human	◎	◎	◎	◎	◎	[104,125,164,165,166]

◎: Exists. ○: Something primitive exists. NE: Does not exist.

**Table 5:** Comparison of acquired immune systems among aquatic organisms.

Phylum	Class		Examples	Acquired Immunity						References
				Lymphoid Tissue						
				Primary		Secondary				
				Thymus	bone Marrow	Spleen	Lymph node	INTESTINE	Kidney and Liver	
Invertebrate	crustacean		crab, shrimp	NE	NE	NE	NE	NE	NE	
	shellfish		oyster	NE	NE	NE	NE	NE	NE	
Vertebrate	jawless fishes	cyclostomes	hagfish	NE	NE	NE	NE	NE	NE	
			lamprey eel	NE	NE	NE	NE	NE	NE	
	jawed fishes	cartilaginous fishes	sharks, rays	◎	NE	◎	NE	◎	◎	[83,167,168]
		osteichthyans	rainbow trout, medaka, carp	◎	NE	◎	NE	◎	◎	[62,103,169]
		amphibian	newt	◎	NE	◎	NE	◎	◎	[161,170,171,172]
			frog	◎	◎	◎	●	◎	◎	[98,173,174,175]
		mammal	seals, dolphin, mouse, human	◎	◎	◎	◎	◎	◎	[24,63,64,105,107]

◎: Exists. ○: Something primitive exists. ●: Exists in some of them. NE: Does not exist.

**Table 6:** Comparison among aquatic organisms of antigen-specific molecules in acquired immune systems.

Phylum	Class		Examples	Acquired Immunity				References
				Antigen-Specific Molecule				
				IgM	IgG/Y	TCR	MHC Class I and II	
Invertebrate	crustacean		crab, shrimp	NE	NE	NE	NE	
	shellfish		oyster	NE	NE	NE	NE	
Vertebrate	jawless fishes	cyclostomes	hagfish	NE	NE	NE	NE	
			lamprey eel	NE	NE	NE	NE	
	jawed fishes	cartilaginous fishes	sharks, rays	◎	● *	○	◎	[83,158,176,177]
		osteichthyans	trout, carp	◎	●	◎	◎	[19,62,178,179,180]
		amphibian	newt	◎	●	◎	◎	[181,182,183]
			frog	◎	◎ **	◎	◎	[184,185,186]
		mammal	Mouse, human	◎	◎	◎	◎	[187,188,189,190]

◎: Exists. ○: Something primitive exists. ●: Exists in some of them. NE: Does not exist. * There are IgX/IgR. ** IgY and IgX are present in African clawed frogs. Ig: immunoglobulin [143].

## 5. Can Immunotoxicological Studies Assess the Effects of Chemicals on Ecosystems?

### 5.1. Current Issue of Ecological Risk Assessment of Chemicals

Current chemical risk assessment is using a single chemical dataset including toxicity, and environmental chemical safety is estimated using the concept that a predicted environmental concentration of chemical (PEC) should not beyond a predicted no-effect concentration (PNEC). Although it seems reasonable at first glance, aquatic organisms meet simultaneously numerous anthropogenic chemicals in the aquatic environment. Indeed, the cumulative impact of several stressors may differ markedly from the impact of the single stressors and can result in nonlinear effects and ecological surprises [191]. The approach of chemical risk assessment is developed from a point of view of management of man-made chemicals for a government. This may be a reason to use a single data set using three trophic level species (i.e., algae, daphnia, and fish) for ecological risk assessment although the tested species are not always representative of all aquatic ecosystems. It appears that a simple concept of a ratio of PEC to PNEC may not be appropriate. Biological impacts of pollution chemicals are depended on the susceptibility of the species to which exposed in the aquatic environment. It is known that the susceptibility of many species fits into a lognormal distribution known as Species Sensitivity Distribution (SSD) [192]. Using SSD dataset, it is available to estimate a ratio of species impacted by pollution chemicals. A ratio of species impacted is recognized as an indicator of an impact for biodiversity (species diversity) and useful for quantitative risk assessment of pesticides [193]. To preserve species diversity in the aquatic ecosystem, HC5 (5% Hazardous Concentration), which means an equivalent chemical concentration that 5% of species would be impacted (95% of species would be preserved), has been defined as no-effect concentrations [194,195,196] and has been considered reasonable for environmental safety in case of pesticides [197,198,199,200]. Recently a new concept called adverse outcome pathway (AOP), which is a structured representation of biological events leading to adverse effects, was discussed and considered relevant to risk assessment [201]. AOP includes the biological impact of the pollution chemical from macro-molecular interactions, cellular, organ, organization, and then reach to population responses. AOP would provide us information on detailed molecular toxicological mechanisms. It would be rather clinical information but still not enough ecologically diagnosing and predicting. To understand ecosystems being polluted by chemicals and predict ecological sustainability, there is a need for studies on impacted and survived organisms (biological properties) rather than studies of conventional lethal concentrations (stressor properties).

### 5.2. Contribution of Immunotoxicological Studies to Ecological Risk Assessment in Population Level

The results obtained from immunotoxicological studies inform us of the presence or absence of exposure to specific or non-specific chemicals that exhibit immunotoxicity and the extent to which an organism’s immune responsiveness and immune function are modified or disrupted by that exposure. Furthermore, there is an expectation that those results may enable the prediction of changes in pathogen susceptibility [99,102,113]. Contributions of immunotoxicological studies would have two directions. The one is expected to take advantage of the high homology of aquatic vertebrates with humans. For example, in drug-discovery research, zebrafish are routinely used as model organisms for preclinical screening of drug efficacy and target validation [202]. The other one is the application of the information for diagnosing and predicting multiple stressor impacts in the aquatic ecosystems, furthering the prediction of ecological sustainability.

**Figure 1:**
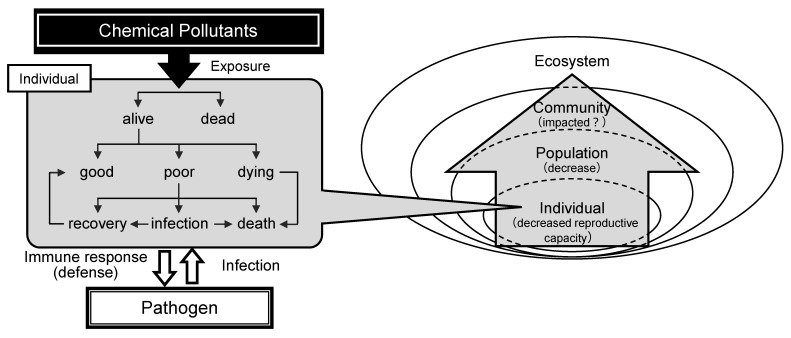
Immunotoxicological effects and ecological risks of chemical pollutants. An individual organism exposed to chemical pollutants will be either alive or dead (**left panel**). Even if the organism is alive, its health will be altered as a function of the degree of exposure (chemical concentration and/or exposure time). Poor health means decreased immune function due to exposure to chemical pollutants and increases the risk of infection by pathogens. If organisms do not recover from damages caused by chemical pollutants, such exposure will consequently cause an increase of death rates and a decrease of the population (**right panel**). The impact may extend from the population to the community and then lead to an alteration of the ecosystem.

Although the number of scientific studies is small, there have been reports that strongly suggest an increase in the risk of infection caused by a decrease in immune function due to exposure to chemical substances [70,110,203]. To understand the increase in the infection risk and ecological risk through immune-dysfunction by chemical exposure, the studies such as a combination of AOP-type and SSD-type studies may be needed. Although this review has given many examples of immunotoxic effects in vitro and in vivo, there is not enough evidence to explain population-level damage. The ecotoxicological endpoint of the adverse effects of chemicals on the immune system would be an increased risk of infection of individual organisms and a consequent decrease in the population. All toxic effects of pollution chemicals in the ecosystem do not end only with the death of the individual. They may cause the extinction of the species with the loss of the population. The aspect of toxicity expression revealed by immunotoxicological research is only one of the many biological consequences of “individual organisms vs. chemical substances”, and it does not allow prediction of the impact of the adverse effects of immunotoxic chemicals on populations of organisms and the consequences for ecosystems. The ecotoxicological focus of immunotoxicological research has been the toxicological effects on the immune defense function, which is one of the roles of the immune system. Knowledge of those effects has made possible consideration of the associated impacts on death rates and decreases of population sizes, including the assessment of the risk of extinction (Figure 1). 

As already mentioned, to estimate the effects of immunotoxicity in population-level ecosystems, there is a need for ecological and immunotoxicological studies on alive but impacted and survived organisms. Then, for the question of whether the ecological risk cannot be evaluated until sufficient immunotoxicological research results are obtained, we have one proposal. An ecosystem includes collections of populations of species that function as an ecological unit. It is well-known that rates of reproduction are taken into account when assessing the risk of extinction [35]. If the number of offspring per capita of a biological population falls below 1, the population will decrease, and there is an increased risk of extinction. Using the rate of reproduction, to evaluate the impact of chemicals on populations of the freshwater fish medaka (*Oryzias latipes*), there were two reports to estimate *r*, a summary index that represents the ability of each population to proliferate. The index *r* can estimate by fitting the life table data for each exposure treatment to the Euler–Lotka equation [35]. Kashiwada et al. tested the later-life effects of neurotoxic insecticide carbaryl at sublethal concentrations in embryos and post-hatch larvae of the medaka and then reported that only in the case of larvae, medaka showed a significant reduction of the population growth rate (*r*) [36]. In addition, Kataoka et al. investigated the effects of silver nanoparticles on the population growth rate of medaka and estimated the extinction time using *r* [37]. Thus, the rates of reproduction (population growth rate) are available for ecological risk assessment of pollution chemicals. If there were AOP-type studies on immunotoxicology and population growth, immunotoxicology would contribute to ecological risk assessment of how immunotoxic effects impact on population levels.

When extrapolating immunotoxicology studies to ecological risk assessment, more complex conditions may need to be considered. In general, an infection becomes established when a pathogen overwhelms the defenses of the host organism. In addition, various environmental factors (e.g., pH, temperature, ultraviolet radiation, and nutritional status of the host), as well as the concentrations of chemical pollutants, influence the outcome of host-pathogen interactions [102,113]. The presence of organisms more sensitive ecologically to pathogens greatly affects the outcome of host-pathogen interactions; it is, therefore, difficult to simply extrapolate the results of immunotoxicological tests conducted in an artificially prepared, unnatural environment (e.g., laboratory conditions) for purposes of ecological risk assessment. Vice versa, standardization of test methods is necessary to obtain reliable data. Most immunotoxicological biomarkers are rather qualitative. The lack of biomarkers that quantitatively evaluate immune function also contributes to the difficulty of predicting effects on ecosystems. The report of hormesis (suppression/inhibition by a chemical at high concentrations and beneficial effects at low concentrations) in oysters [139] means that it is possible to misjudge effects by simply and independently evaluating biomarkers such as the phagocytic ability of phagocytic cells, ROS, antibody production, and other biomarkers in the immunotoxicological evaluation of environmental pollutants. Immune responses are reflected by individual genotypic and phenotypic properties and simultaneously depend on ambient environmental factors including pathogens and toxic chemicals [33]. Hence, comprehensive research of immunotoxicological biomarkers is necessary to predict the ecological impact of immunotoxicological effects at the individual level and for ecological risk assessments at the population level.

## 6. Conclusions

Immunotoxicological studies have very effectively enabled the prediction of the effects of chemical exposure on susceptibility to infections by pathogens. Although the effects of that exposure may not be exhibited by acute toxicity, studies of the chronic effects may enable a quantitative assessment of increased mortality due to infection and decreased population sizes caused by chronic biological effects (e.g., increased rate of pathogen infection or decreased rate of population growth due to decreased functionality of the immune system) resulting from, inter alia, adverse impacts on the immune system. Anthropogenic chemicals are found today even in polar regions, where there is relatively little human activity, and aquatic organisms are unwittingly exposed to those chemicals. Immunotoxicological studies using aquatic organisms as models may be a new tool for assessing the risks to aquatic ecosystems associated with the presence of toxic chemicals. Furthermore, the combined study of ecotoxicology and immunotoxicology is expected to become a new field of study for assessing the ecological effects of chemical pollutants by assessing the health of aquatic organisms exposed to those chemicals.
